# Supplementary material for: Enhanced spin Seebeck effect signal due to spin-momentum locked topological surface states
Source: Nat Commun. 2016 May 4;7:11458. doi: 10.1038/ncomms11458 (PMC4857385; doi:10.1038/ncomms11458)
Supplement: Supplementary Information — Supplementary Figures 1-7 [file ncomms11458-s1.pdf]

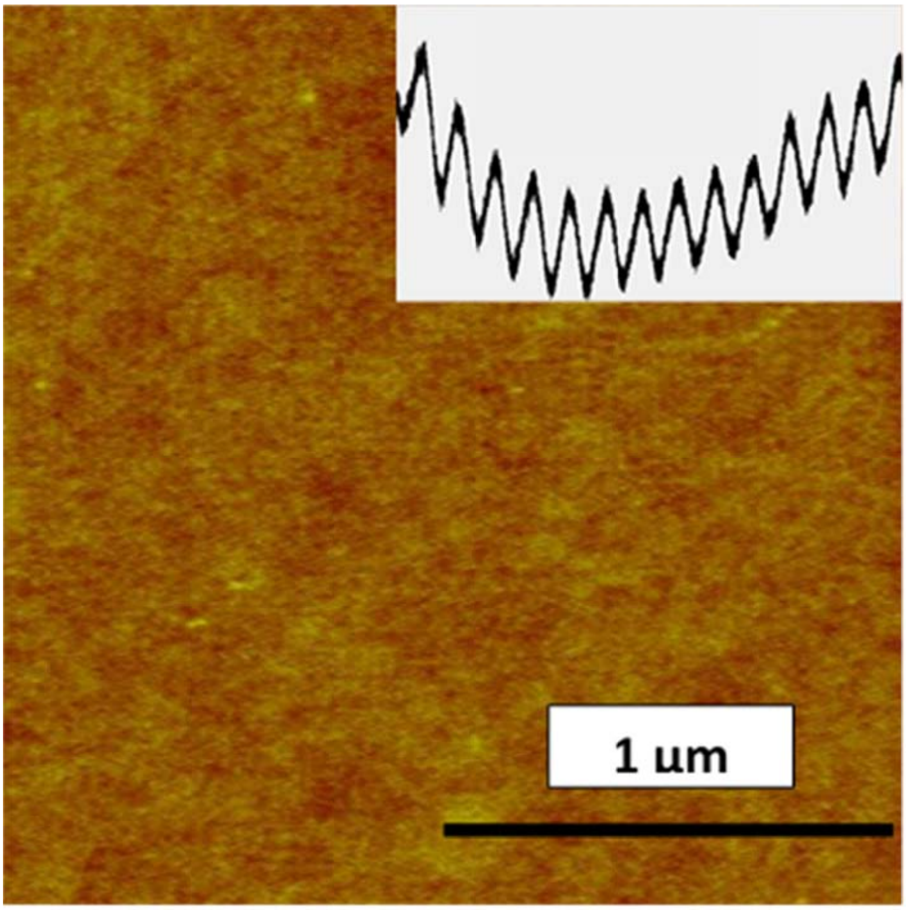

Supplementary Figure 1. AFM image (2 μm × 2 μm) of a representative YIG (111) thin film. The 20 nm thick YIG films are atomically flat with a typical rms roughness ~ 0.12 nm. Flat YIG surface is important for high quality TI films. Prior to growth, the substrate is heated (600 °C for 30 min) to remove organic contamination. The inset shows the RHEED intensity oscillations during YIG growth.

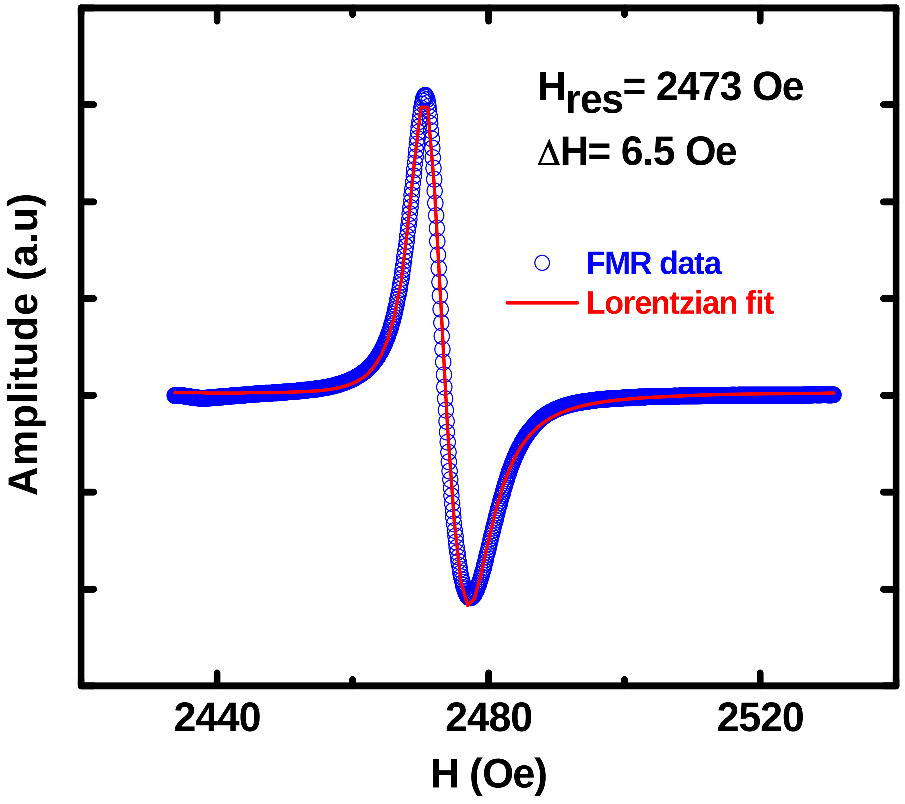

Supplementary Figure 2. Typical FMR spectrum for 20 nm thick YIG films on GGG (111). In the FMR spectrum taken at 9.6 GHz, the blue circles are the experimental data and the red line is a Lorentzian fit. The FMR linewidth (ΔH) obtained from the fit is as narrow as 6.5 Oe. The measured resonance field (H_{res}) yields $4\pi M_s \sim 2200$ Oe from the Kittel formula.

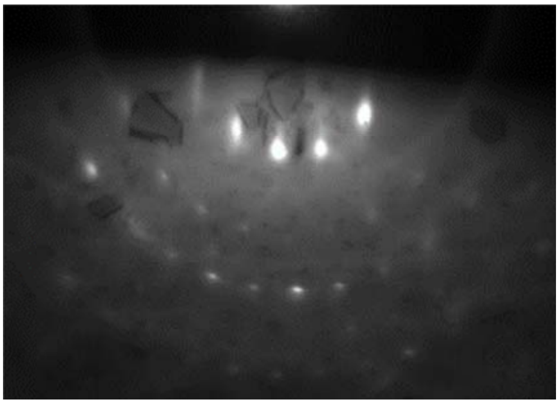

a, YIG (111)/ GGG substrate after deposition in PLD

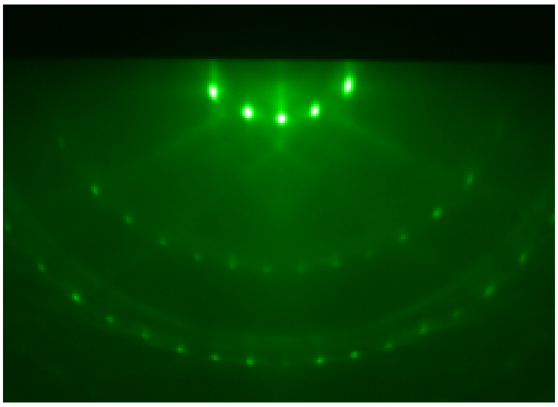

b, YIG (111)/ GGG substrate prior to TI growth in MBE

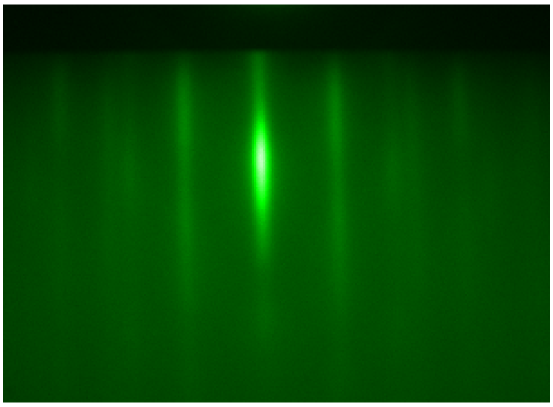

c, 5 QL $(Bi_{0.24}Sb_{0.76})_2Te_3$ grown on YIG

Supplementary Figure 3. RHEED patterns of YIG substrate and TI film on YIG. a, YIG film is epitaxially grown on GGG as indicated by the RHEED pattern taken in the PLD chamber. **b,** RHEED pattern taken on YIG prior to TI growth in the MBE chamber excludes the possibility of degradation during sample transfer and annealing. **c,** RHEED pattern of 5 QL $(Bi_{0.24}Sb_{0.76})_2Te_3$ grown on YIG indicates very flat TI surface and high quality crystalline TI film.

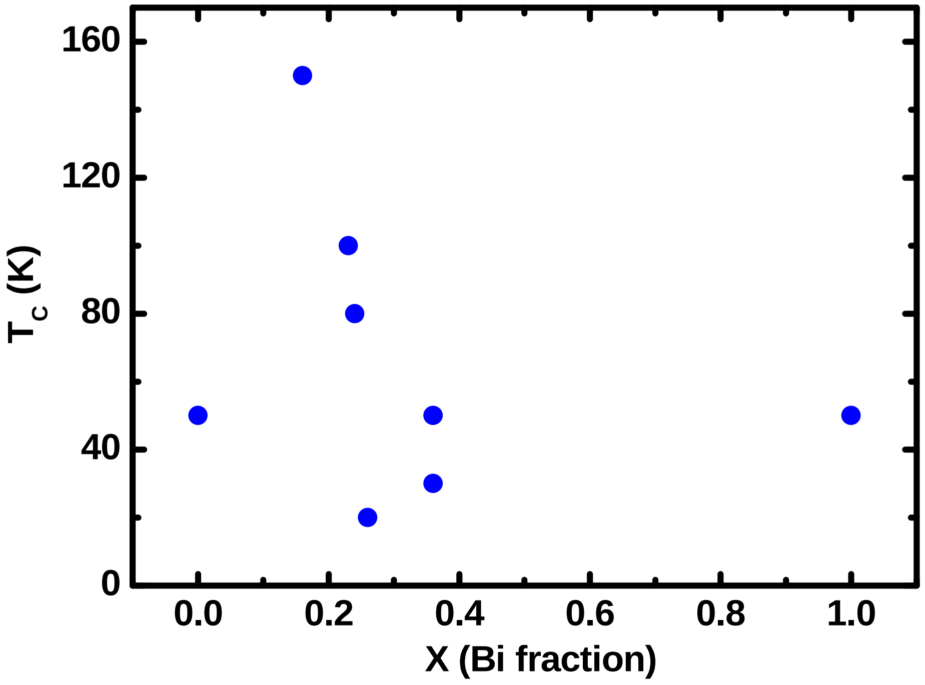

Supplementary Figure 4. Curie temperature (T_C) of the ferromagnetic TI surface vs. Bi fraction. T_C of induced ferromagnetic TI surface was determined from the AHE measurements in samples with various Bi fractions. T_C is not obviously correlated with the Bi fraction/carrier concentration. The ferromagnetic transition is far below 300 K at which the SSE experiments were conducted.

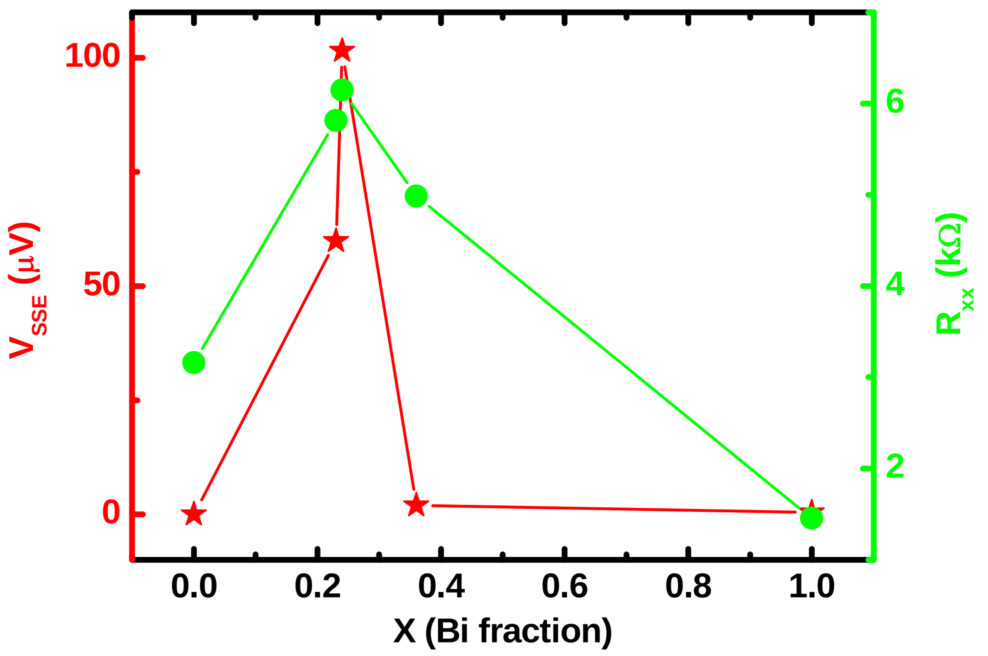

Supplementary Figure 5. V_{SSE} and corresponding R_{xx} vs. Bi fraction. The left (V_{SSE}) and right (R_{xx}) axes denote the SSE voltage and corresponding longitudinal sheet resistance respectively for heterostructure samples with different x's. As Bi fraction is decreased from 1 to 0.24, V_{SSE} shows an abrupt change by a factor of 200, while R_{xx} only increases by a factor of 4.

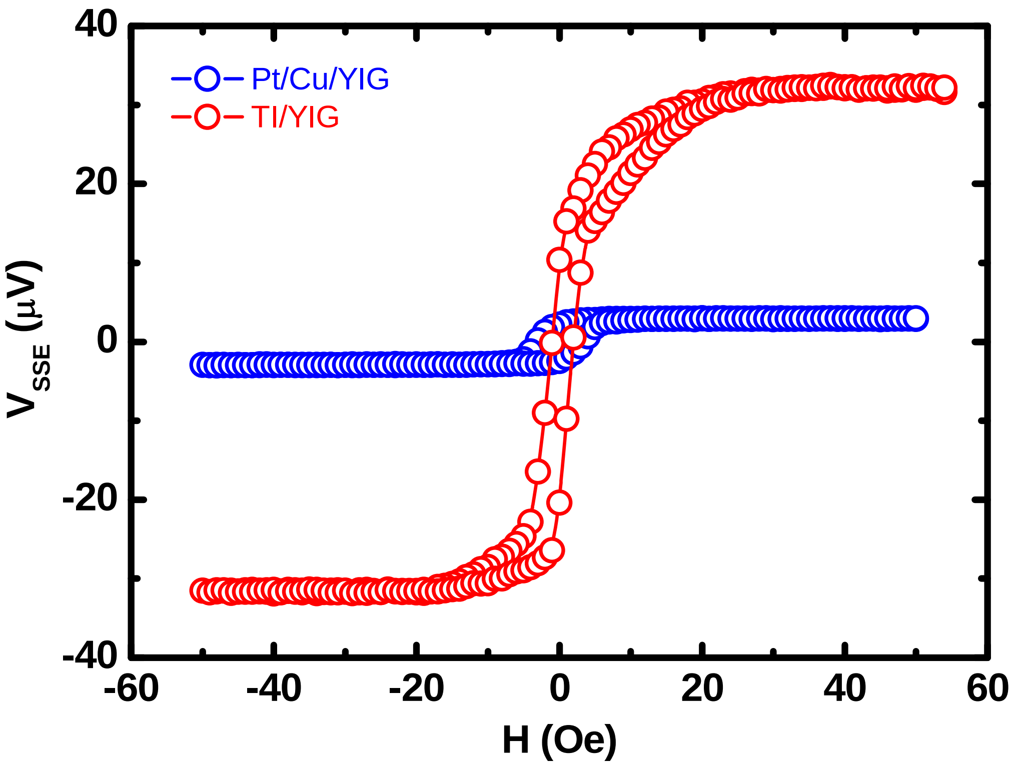

Supplementary Figure 6. A comparison between SSE signals from Pt/Cu/YIG and TI/YIG devices. In Pt/Cu/YIG, a 3 nm Cu spacer layer is inserted between a 5 nm thick Pt and YIG to suppress the proximity effect induced anomalous Nernst contribution. The other device consists of 5 QL $(Bi_{0.24}Sb_{0.76})_2Te_3$, the most insulating TI and YIG. The SSE voltage generated by TI/YIG is one order of magnitude larger than that by Pt/Cu/YIG.

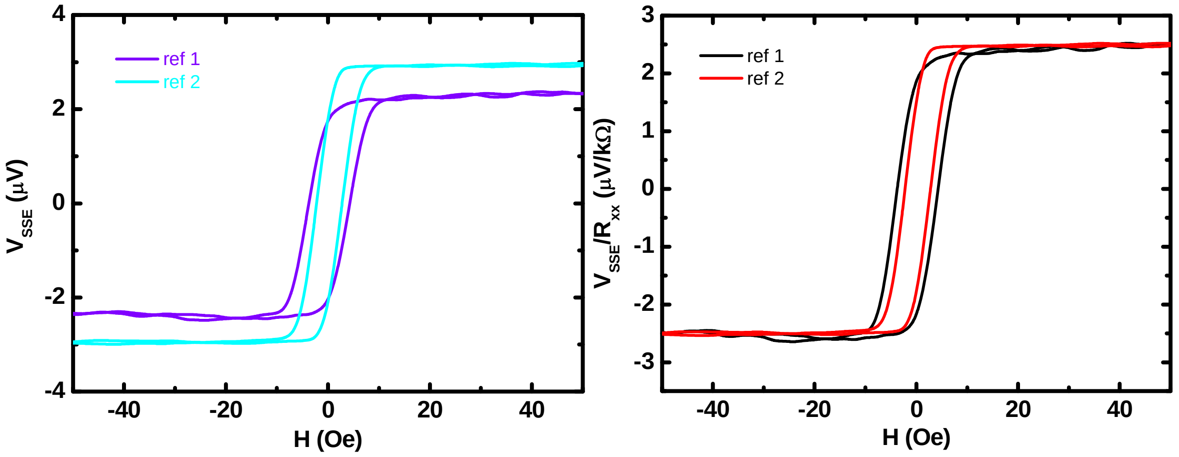

Supplementary Figure 7. Comparison between SSE signals from two Pt/YIG samples. Two Pt (5 nm)/YIG (20 nm) samples were fabricated at different times. YIG was grown by PLD, while Pt was deposited by sputtering in a different chamber. The fabrication requires breaking the vacuum and transfer in air after YIG growth. These two samples (ref 1 and ref 2) have the same nominal device geometry and were measured under the same conditions. The SSE voltage shows a variation of ~20% in magnitude; however V_{SSE}/R_{xx} only differs by less than 5%. These results indicate the interface quality in these samples does not vary significantly to cause large variations in SSE magnitude.
